# Kinetics Analysis and Susceptibility Coefficient of the Pathogenic Bacteria by Titanium Dioxide and Zinc Oxide Nanoparticles

**DOI:** 10.15171/apb.2020.007

**Published:** 2019-12-11

**Authors:** Mahmood Alizadeh-Sani, Hamed Hamishehkar, Arezou Khezerlou, Mohammad Maleki, Maryam Azizi-Lalabadi, Vahid Bagheri, Payam Safaei, Taher Azimi, Mohammad Hashemi, Ali Ehsani

**Affiliations:** ^1^Student’s Scientific Research Center, Food Safety and Hygiene Division, Environmental Health Department, School of Public Health, Tehran University Of Medical Sciences, Tehran, Iran.; ^2^Students Research Committee, Department of Food Sciences and Technology, Faculty of Nutrition and Food Sciences, Tabriz University of Medical Sciences, Tabriz, Iran.; ^3^Drug Applied Research Center, Tabriz University of Medical Sciences, Tabriz, Iran.; ^4^Department of Food Hygiene and Aquaculture, Ferdowsi university of Mashhad, Mashhad, Iran.; ^5^Department of Food Science and Technology, Faculty of Agriculture, University of Tabriz, Tabriz, Iran.; ^6^Department of Microbiology, School of Medicine, Shahid Beheshti University of Medical Sciences, Tehran, Iran.; ^7^Department of Pathobiology, School of Public Health, Tehran University of Medical Sciences, Tehran, Iran.; ^8^Department of Nutrition, Faculty of Medicine, Mashhad University of Medical Sciences, Mashhad, Iran.; ^9^Nutrition Research Center, Department of Food Sciences and Technology, Faculty of Nutrition and Food Sciences, Tabriz University of Medical Sciences, Tabriz, Iran.

**Keywords:** Pathogenic bacteria, ZnO, TiO2, Kinetics, Susceptibility coefficient

## Abstract

***Purpose:*** The increase of bacterial resistance to common antibacterial agents is one of the major problems of health care systems and hospital infection control programs. In this study, antimicrobial activity of titanium dioxide (TiO_2_ ) and zinc oxide (ZnO) nanoparticles (NPs) was investigated against *E. coli, Salmonella enteritidis*, *Listeria monocytogenes*, and *Staphylococcus aureus* pathogenic bacteria by determining sensitivity coefficient and kinetics of bacterial death.

***Methods:*** Antimicrobial tests were performed with ~10^6^ CFU/mL of each bacterium at baseline. At first, minimum inhibitory concentration (MIC) was concluded by the dilution method and then, death kinetic and susceptibility coefficient of NPs suspensions was determined at 0 to 360 min. treatment time.

***Results:*** The results of this study revealed that, the highest susceptibility was observed for *L. monocytogenes* (Z=0.025 mL/μg) to TiO_2_ NPs, whereas the lowest susceptibility was obtained in the reaction of ZnO NPs with *S. enteritidis* (Z=0.0033 mL/μg). The process of bacterial death in NPs suspension was assumed to follow first-degree kinetic and the survival ratio of bacteria decreased by the increase in treatment time. An increase in the concentration of NPs was seen to enhance the bactericidal action.

***Conclusion:*** Results showed that *L. monocytogenes* had higher sensitivity compared to *S. enteritidis*. The results of this study also demonstrated that TiO_2_ NPs have a strong antimicrobial effect in comparison with ZnO NPs and it could be employed to aid the control of pathogenic bacteria.

## Introduction

Nowadays, new approaches are required to control harmful microorganisms. A broad spectrum of microorganisms is balanced with the human environment and food products; however, the uncontrolled growth of microorganisms can cause serious problems.^1-3^ Food poisoning, foodborne and hospital infections are some of the oldest health care problems with challenging control programs.^4-6^ Recent studies have shown that some metals/oxides such as CaO, MgO, as well as many nanoparticles (NPs) such as Ag, zinc oxide (ZnO), CuO, and titanium dioxide (TiO_2_) are known to have marked antibacterial activities.^7-9^ Nanoparticles have received considerable attention as antimicrobial agents, which can substitute common antimicrobial agents, such as antibiotics and chlorine disinfectant in order to control spoilage and the spread of pathogenic bacteria in food and in the environment such as hospitals.^10,11^

In recent years, nanotechnology has been increasingly applied in different fields, especially in medical, food and pharmaceutical domains.^12-14^ Among various NPs, TiO_2_ and ZnO are widely used due to their strong antimicrobial effects. These materials, when synthesized on a nanoscale, exhibit strong antimicrobial effects because of an increase in surface-to-volume ratio and their specific surface area.^15,16^ It has been suggested that these nanomaterials react with proteins especially with -SH groups; consequently, which leads to protein inactivation.^11,15^ Several studies have reported the antimicrobial effects of various NPs such as silver and copper against *E. coli* and *Bacillus subtilis,*^17^ silver NPs against multidrug-resistant bacteria^18^ and TiO_2_ NPs against pathogenic bacteria.^8,19^ According to the results obtained by various tests such as proteomics research, transmission electron microscopy (TEM) and scanning electron microscopy, it is proposed that the mechanism of antimicrobial activity varies from one nanoparticle to another, because NPs react with important elements of bacterial membrane and cell wall, which cause structural change and damage, destruction of the proton motive force, and finally cell death.^11,20,21^ Nano-antimicrobial agents can be used in coating surfaces in order to produce antimicrobial characteristics in food and in medical devices and water treatment filters.^22^

The effectiveness of nanomaterials’ antimicrobial activity is determined by experimental techniques that measure microorganism viability after exposure. In industrial functions of antimicrobial agents, numerical and mathematical models and quantitative parameters are essential for design optimization, performance assessment and life-time prediction of antimicrobial techniques. Therefore, for the application of nanomaterials in commercial and industrial scales, the numerical models and quantitative parameters are necessary to determine the efficiency, design optimization, and survival rate prediction of antimicrobial agents.^17,23^ As two of quantitative parameters, susceptibility coefficient and death kinetic have been applied in numerical models to evaluate the antimicrobial effects of nanomaterials against foodborne microorganisms.^24,25^

The main objective of this study was to evaluate the antimicrobial activity of TiO_2_ and ZnO NPs against *E. coli*, *S. enteritidis*, *L. monocytogenes*, and *S. aureus* pathogenic bacteria, and also to determine the susceptibility coefficient and death kinetic using predictive modeling of microbial growth.

## Materials and Methods

This study was conducted as an empirical research in the laboratory. In order to investigate the antibacterial activity, commercial ZnO and TiO_2_ with the purity of 99% were purchased from US Research Nanomaterials, Inc., Houston, U.S.A. Pathogenic bacterial strains *E. coli* (ATCC-25922), *Salmonella enteritidis* (ATCC-49221), *Listeria monocytogenes* (ATCC-13932) and *Staphylococcus aureus* (ATCC-33591) were obtained from Iranian Biological and Genetic Resources Center, Tehran, Iran. All the applied reagents were of analytical grade.

### Preparation of nanoparticles suspension

The TiO_2_ and ZnO NPs suspensions were prepared in various concentrations (0.25, 0.5, 1, 1.5, 2, 2.5, 3, 3.5, 4, 4.5, 5, 6, 7, 8, 9 and 10 mg/mL) of each nanoparticle in sterile distilled water. Then, an ultrasonic probe sonicator (UCD-1200, Bio-Base, Shandong, China) was applied for 15 min. with 150W, and Ultra-Turrax (IKA, Germany) homogenizer was used for 10 min. with 10 000 rpm in order to well-disperse NPs.^8^

### Bacterial culture and preparation of bacterial suspension

Standard bacterial strains included gram-negative bacteria (*E. coli* and *S. enteritidis*) and gram-positive bacteria (*L. monocytogenes* and *S. aureus*), were exploited as tested bacteria and kept under freeze-dried conditions. Then, standard strains of the bacteria were cultured at 37°C for 24 h on a Trypticase Soy agar (TSA) medium (Merck, Germany). After incubation, the bacterial single-colonies were collected from medium by sterile loop, and used to prepare bacterial suspensions.

To prepare a bacterial suspension, at first, bacterial single-colonies were added to 1 mL phosphate buffer saline (PBS), its turbidity was then adjusted to 0.5 McFarland standard with ~1.5 × 10^8^ CFU/mL and diluted to the desired bacterial density (~1.5 × 10^6^ CFU/mL). The turbidity approved by measuring the absorbance of bacterial suspension using an ultraviolet (UV) spectrophotometer (UNICO-2100, Northbrook, Illinois, USA), in ranging of 0.08 to 0.1 at 625 nm.^8,26^

### Kinetics of bacterial death

At first, to kinetic of bacterial death, minimum inhibitory concentration (MIC) for each of the bacteria in contact with NPs suspension was determined as previously described with some modification.^27-29^ MIC is generally defined as the lowest concentration of an antimicrobial agent that inhibits the visible growth of a microorganism after overnight incubation. Then, the suspensions of the desired microorganisms (20 μL) with different concentrations of ZnO/TiO_2_ NPs (0.25, 0.5, 1, 2, 3, 4, 5, 6, 7, 8, 9, and 10 mg/mL) (20 μL) were added to microplates containing 160 μL broth neutralizing medium (Trypticase Soy broth). The microplates containing NPs and bacteria were incubated for approximately 24 h in a shaking incubator (100 rpm, temperature of 37°C).

Two concentrations of bacterial suspension (1 and 2 × MIC) were added to the medium containing NPs and were then incubated at 37°C for 24 h. At the desired time (from 0 to 360 min), a bacterial/nanoparticle suspension was sampled and spread on TSA plates. The number of colonies were counted and recorded for each time, bacteria and concentration.

Survival rates (N/N_0_) were calculated by dividing the number of colonies at the time of the sampling (N) by the number of colonies at the time when they had no contact with ZnO/TiO_2_ NPs suspensions (N_0_). To study the kinetic of bacterial death, the kinetic of first-degree death was used. The general form of this kinetic is as follows:^7,30-32^

(1) dNdt=−kN0

Where k is the death rate constant, N_0_, the number of initial bacterial colonies, and N is the number of bacterial colonies at the time t. In this study, the sensitivity of NPs (Z) based on mL/μg, is obtained by the following equation:^17,32^

(2) $$Z=\frac{-\ln(\frac{N}{N_{0}})}{C}$$

In this equation N is the bacterial colony-forming units (CFUs) on the agar plate containing NPs, N_0_ is the CFUs on the agar plate, and C is the concentration of NPs (mg/mL). By the use of Z and C values, the survival fraction (N/N_0_) can be predicted. A higher Z value means that the bacteria are more sensitive to NPs, which also indicates that NPs have more antimicrobial susceptibility (stronger antimicrobial properties).

### Statistical analysis

Statistical analysis was carried out using SPSS software (version 16.0, IBM; Armonk, USA), with Descriptive statistics and Independent-Samples *t* test analysis. Differences were determined to be significant at *P* ˂ 0.05. All tests of the present study were performed in triplicate.

## Results and Discussion

**Figure 1:**
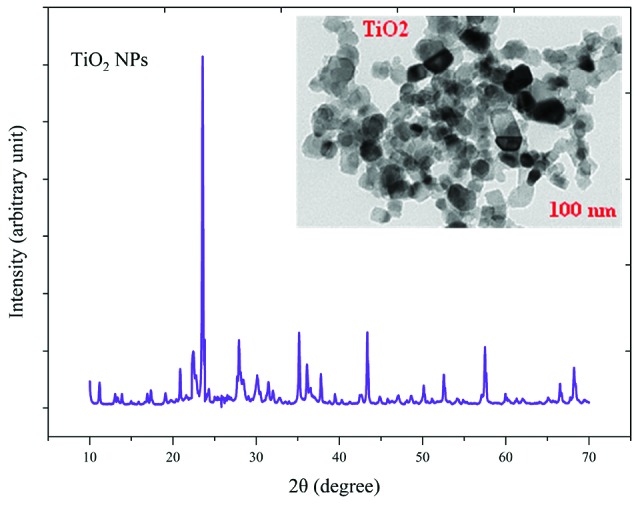
X-Ray diffraction (X-RD) spectra and Transmission Electron Microscopy (TEM) image of TiO_2_ nanoparticles.

**Figure 2:**
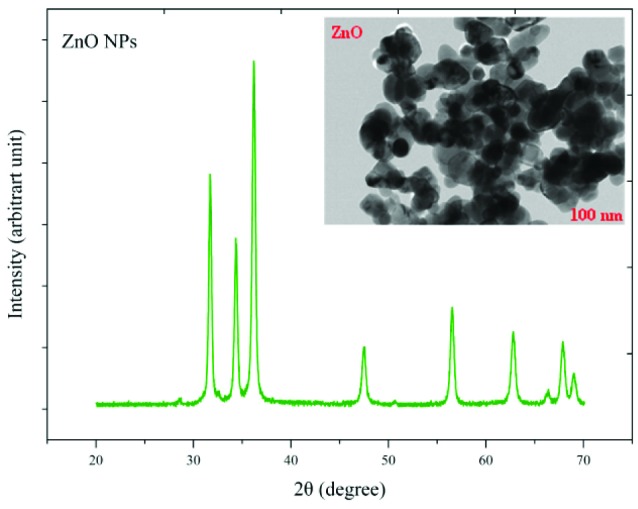
X-Ray diffraction (X-RD) spectra and Transmission Electron Microscopy (TEM) image of ZnO nanoparticles.

The crystalline state and the presence of impurities in NPs were identified by X-ray diffraction (XRD) and TEM (Figures 1 and 2). TEM results showed that NPs have a hexagonal and cubic (ZnO) andtetragonal (TiO_2_) shape and the diameter of particles was about ~20 nm and 10~25 nm for ZnO and TiO_2_ NPs, respectively. The specific surface area, by measuring the adsorption isotherms of N_2_ at −196°C (BET; Belsorp-mini), was approximate ~90 m^2^/g and ~185 m^2^/g for ZnO and TiO_2_ NPs_,_ respectively.

According to the obtained results in Table 1, that shows the antimicrobial activity of ZnO and TiO_2_ NPs suspensions against the tested bacteria, MIC values for gram-negative and gram-positive bacteria to ZnO NPs were found to be ~2.5-3 mg/mL and ~1.5-2 mg/mL, respectively. Also, MIC values for gram-negative bacteria to TiO_2_ NPs were found to be ~2-2.5 mg/mL and for Gram-positive bacteria was ~1-1.5 mg/mL.

**Table 1:** MIC of TiO2 and ZnO NPs against pathogenic bacteria

**Bacteria strains**	**MIC (mg/mL)**	
	**ZnOTiO**_2_	
	**Mean±SD**	**Mean±SD**
*E. coli*	2.50±0.18	2.00±0.33
*S. enteritidis*	3.00±0.21	2.50±0.17
*S. aureus*	2.00±0.17	1.50±0.19
*L. monocytogenes*	1.50±0.15	1.00±0.14

MIC: minimum inhibition concentration; TiO2 NPs: titanium dioxide nanoparticles; ZnO NPs: zinc oxide nanoparticles; SD: standard deviation.

**Figure 3:**
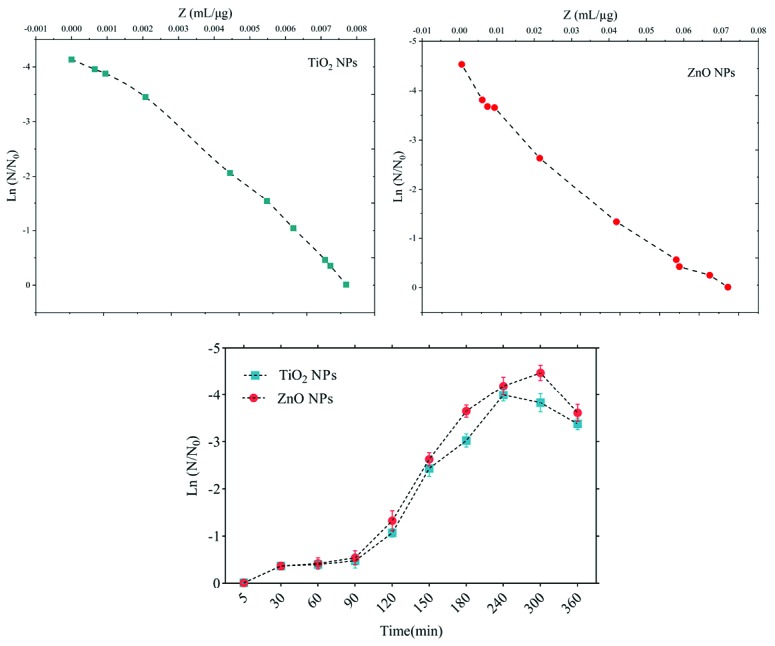
Death kinetics (bacterial population changes) and sensitivity coefficient of *Listeria monocytogenes* bacterium to time.

**Figure 4:**
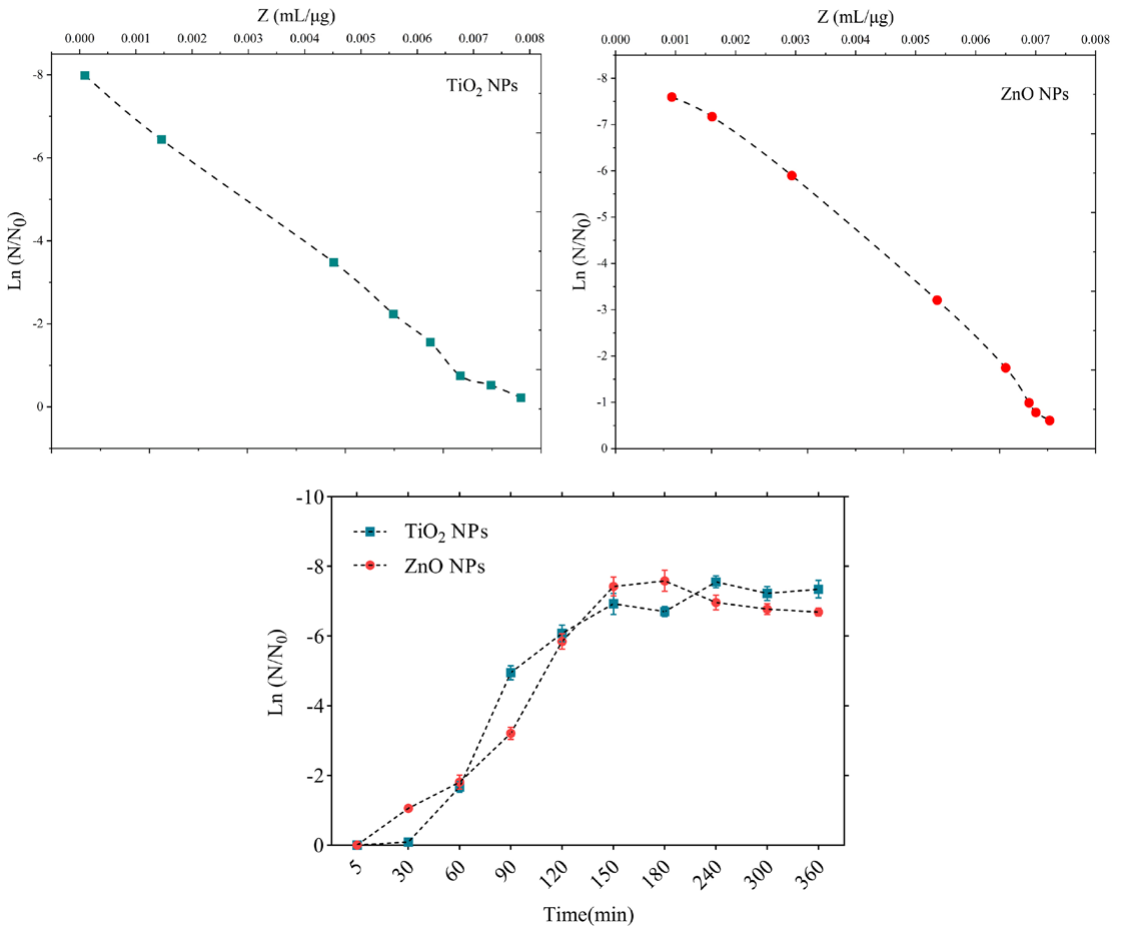
Death kinetics (bacterial population changes) and sensitivity coefficient of *Staphylococcus aureus* bacterium to time.

**Figure 5:**
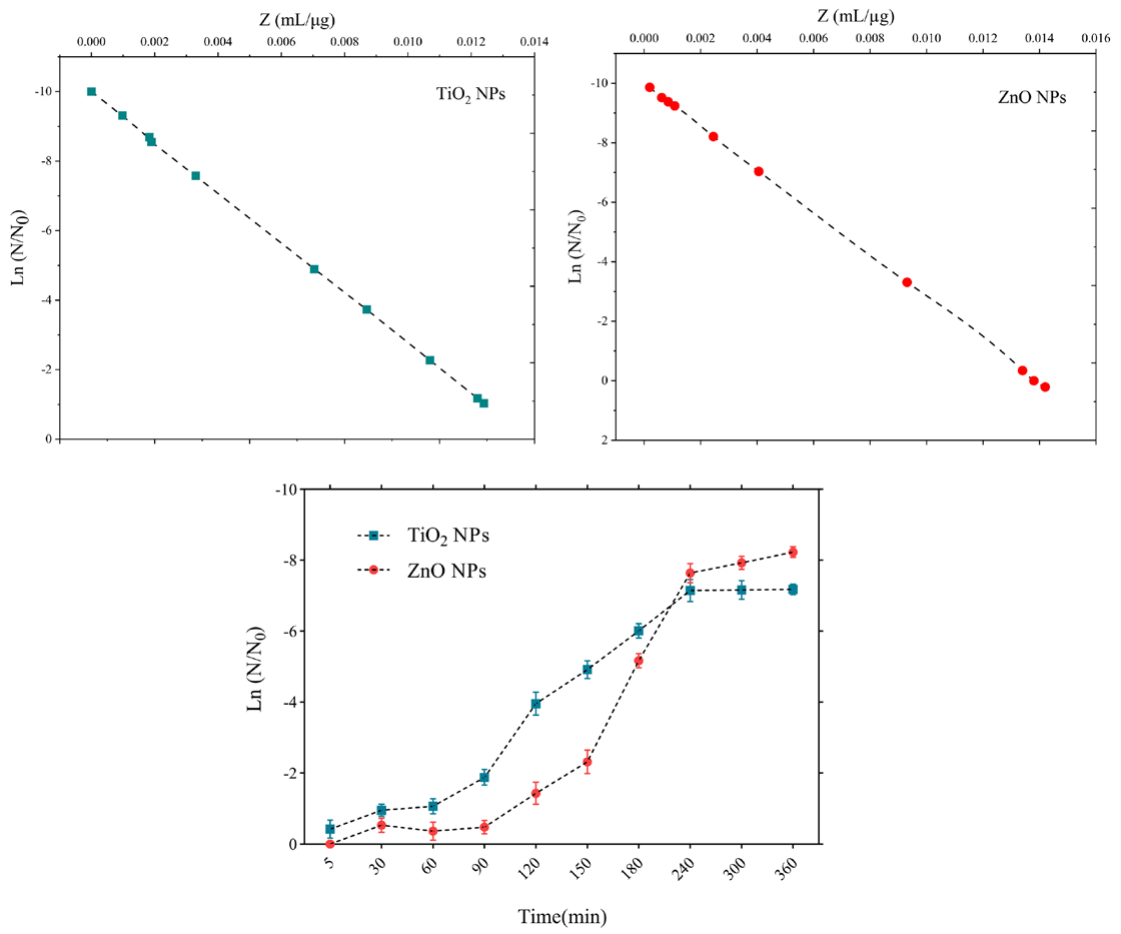
Death kinetics (bacterial population changes) and sensitivity coefficient of *E. coli* bacterium to time.

**Figure 6:**
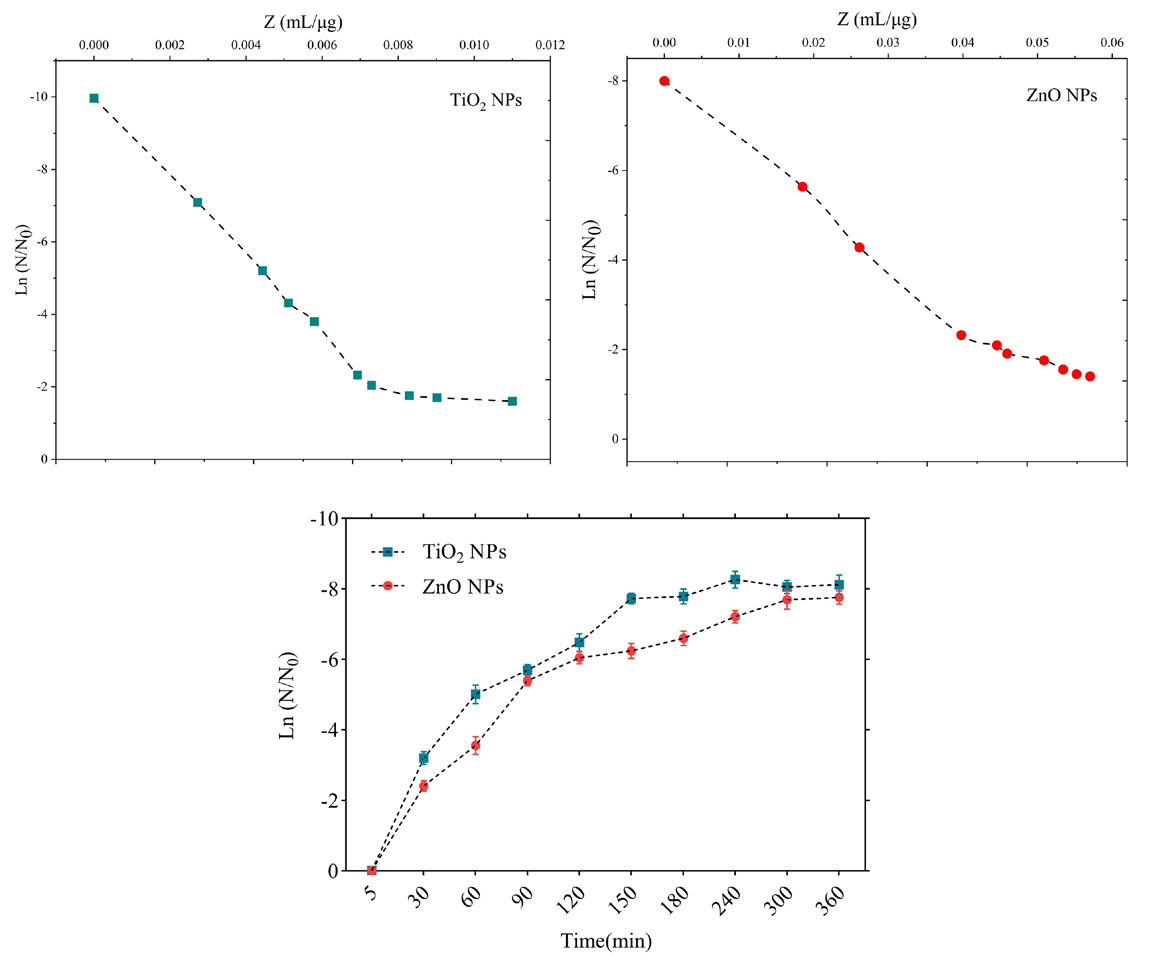
Death kinetics (bacterial population changes) and sensitivity coefficient of *Salmonella enteritidis* bacterium to time.

The sensitivity coefficient of each bacterium to NPs suspension at each sampling period was calculated. The result of the mean of the sensitivity coefficient is shown in Table 2. The sensitivity coefficient of gram-positive bacteria to both NPs has increased by increasing the concentration of NPs from 1 to 2 MIC values, but the average sensitivity coefficient for gram-negative bacteria, especially *S. enteritidis*, has decreased by increasing the concentrations of both NPs. The changes of the bacterial cells in contact with NPs suspension at different times and the kinetics of their death were calculated. The changes in the populations of bacteria and the sensitivity coefficient are shown in Figures 3-6, respectively. The kinetics of bacterial death showed that the survival ratio of each bacteria decreased by increasing concentrations of NPs. The population of bacteria decreased linearly over time. The sensitivity coefficient of both NPs has increased by an increase in contact time for *S. aureus* and *L. monocytogenes*. As shown in Table 2, the sensitivity coefficient values for TiO_2_ NPs are always higher than ZnO NPs. The kinetic results indicated that the population of bacteria declined when the concentration of NPs increased. At a concentration of one-time the MIC value, the re-growth of bacteria was observed. But with increasing concentrations of twice the MIC value, after reducing the population of the bacteria, there was no re-growth.

**Table 2:** The sensitivity coefficient for each of the pathogenic bacteria and nanoparticles studied

**Bacteria strains**	**Z value (mL/µg)**		*P* **value**
	**TiO**_2_ **ZnO**		
	**Mean±SD**	**Mean±SD**	
*L. monocytogenes*	0.025±0.0052	0.019±0.0042	0.033*
*S. aureus*	0.016±0.0041	0.012±0.0033	0.044*
*E. coli*	0.0057±0.0010	0.0048±0.0010	0.078
*S. enteritidis*	0.0045±0.0010	0.0033±0.0010	0.086

MIC: minimum inhibition concentration; TiO2 NPs: titanium dioxide nanoparticles; ZnO NPs: zinc oxide nanoparticles; SD: standard deviation. * P value reported based on ANOVA test (significant at level of *P* value <0.05.)

The average sizes of ZnO and TiO_2_ NPs were ~20 nm and 10~25 nm, respectively. Reducing the particle size of NPs can change their structural and physicochemical properties, also their toxicity would increase due to their access to biological organisms.^7,9,11^ In the XRD profile of ZnO and TiO_2_ NPs, a peak of oxygen was observed. TiO_2_ and ZnO NPs both contain high atom density, known to be highly reactive NPs.^33,34^ The specific surface area of TiO_2_ NPs is approximately 2.05 times greater than the specific surface area of ZnO NPs. The decrease in size and the increase in specific surface area, as the factors in increasing the number of reactive groups on the particle surface, were considered as the most important factors for the increase in the toxicity of NPs and increased antimicrobial activity.^33^ Probably, the increase in reactive groups which act as the active sites for the formation of reactive oxygen species (ROS) including superoxide, hydrogen peroxide, and radical hydroxyl cause oxidative stress.^9,11^ The results showed that gram-negative bacteria were more resistant to NPs compared to gram-positive bacteria. One of the reasons for the lower sensitivity of Gram-negative bacteria can be the fact that external membranes of gram-negative bacteria, such as *Salmonella* and *Escherichia coli*, are predominantly composed of a strong lipopolysaccharide layer (LPS), which is considered to be a permanent barrier against NPs.^35^ The results obtained by Yoon et al proved that *B. subtilis* was more sensitive than *E. coli*.^17^ In the present study, gram-positive bacteria were more susceptible to TiO_2_ NPs rather than ZnO NPs (1 and 1.5 mg/mL inhibitory concentrations versus 2.5 and 3 mg/mL). In addition, the first-degree equation had been used to describe the kinetic of bacterial growth. As seen in Figures 3-6, when the logarithmic ratio of surviving bacteria and the time of exposure are plotted as vertical and horizontal axes, it can be seen that the population of the tested bacteria decreases linearly. The results obtained by Sawai et al are consistent with the results of this section of the present study.^30^ In colony counting, when the bacteria survival ratio is negative over time, it can be concluded that NPs have strong antimicrobial activity.^30,31,36^ As the kinetic charts of bacterial death (Figures 3-6) are deduced to a specific time (common to both concentrations of NPs), the survival ratio decreased rapidly with increasing nanoparticle concentrations. In addition, the antibacterial activity of NPs suspension increased with concentration increase. This result can be associated with higher toxicity at higher concentrations of NPs.^15^ Of course, this cannot indicate the linear relationship between the concentration and antimicrobial potential of NPs, since at higher concentrations there is a potential for bacterial compatibility with NPs. As the contact time increases, with the increase in the concentration of NPs, the survival ratio of the bacteria reduces. In other words, the antibacterial activity of NPs has increased with increasing contact time and concentration of NPs against tested bacteria, which could indicate the broad spectrum of antimicrobial properties of these NPs.^15,37^ Although, their antimicrobial activity against other microorganisms is required. By increasing the concentration of NPs by one-fold to twice the MIC value, the value of the Z parameter for Gram-negative bacteria reduced, which can be due to the compatibility resistance bacteria at higher concentrations.^36^ The slope of log N/N_0_ ratio in Gram-negative bacteria was lower compared to Gram-positive bacteria by increasing the concentration of NPs, which could be due to the resistance of these bacteria to NPs investigated in this study.^32^ Shahverdi et al concluded in their study that a gram-positive bacterium (*S. aureus*) had a higher sensitivity to chemicals than to antibiotics because of the differences in its cell wall composition compared to *E. coli*.^38^ At each concentration of NPs, the sensitivity coefficient was determined using equation (2), and the Z value was calculated for a range of NPs concentration and contact time. The results of the over-time sensitivity analysis and the concentration of NPs showed that the magnitude of the sensitivity of both NPs increased by increasing contact time. Although the magnitude of the sensitivity of both NPs is very close, the value of this parameter has always been higher for TiO_2_ NPs than ZnO NPs. As can be seen in diagrams and Table 2, at a time interval of 300 and 360 minutes, the TiO_2_ NPs sensitivity coefficient was higher. The values of the sensitivity coefficient of gram-positive bacteria for ZnO NPs were lower than that of TiO_2_ NPs, which indicates that *L. monocytogenes* and *S. aureus* are more sensitive to TiO_2_ NPs.

Various studies have reported that Gram-negative bacteria are usually more resistant to antimicrobial compounds. This resistance can be attributed to a more complex cell wall of gram-negative bacteria than gram-positive bacteria.^35,39^ Antimicrobial materials such as NPs have been taken into account because of their strong antimicrobial effects in low concentrations against microorganisms.^40,41^ Usually, all of the metal NPs have the ability to reduce or remove the microorganisms by two main mechanisms: (a) free metal ion toxicity arising from dissolution of metals from the surface of NPs and (b) oxidative stress via generation of ROS, by using hydrogen peroxide (H_2_O_2_) and organic hydroperoxides, on the surface of NPs.^11,35,42^ In fact, NPs can affect the survival of bacteria by agglomeration on the surface of bacteria and changing the structure of lipids, peptidoglycan, proteins, and their DNA.^11^ But there may be a diversity in the effects of NPs on specific types of bacteria. For example, Alizadeh-Sani et al showed the antimicrobial effects of TiO_2_ NPs against *S. aureus, L. monocytogenes, E. coli, P. fluorescence*, and *S. enteritidis*.^8^ In another study, Ruparelia et al demonstrated that copper NPs had a great impact as an antimicrobial agent against *E. coli*, *B. subtilis*, and *S. aureus* than TiO_2_ NPs.^43^ Fu et al investigated the antibacterial activity of TiO_2_ nanocomposites against, *Escherichia coli* (DH 5α) and *Bacillus megaterium* (QM B1551). The results of their study showed that TiO_2_ NPs had good inhibitory effects, especially versus *B. megaterium* (as a gram-positive bacteria).^44^ TiO_2_, as a metal oxide, is known as one of the most widely used semiconductor NPs with specific hydrophilic and photocatalytic properties, which leads to antimicrobial, and UV protecting characteristics.^8,45^ These properties are obtained without the use of chemicals and only by using sunlight and water.^46^ TiO_2_ NPs can produce active oxygen species when exposed to sunlight. Moreover, the antimicrobial activity of TiO_2_ NPs is related to its crystal structure, the kind of artificial light, UV light intensity, shape, and size, as well as the production of ROS active radical species, hydrogen peroxide, superoxide radical, and hydroxyl radical.^11,35,47^ These active species destroy the outer membrane of the bacteria, namely phospholipids, proteins, and lipopolysaccharides, and finally damage the bacteria. In addition, several studies reported the antibacterial activities of ZnO NPs against foodborne pathogens including *E. coli, L. monocytogenes*, *Salmonella,* and *S. aureus*.^48^ The antimicrobial effects of ZnO NPs are usually related to the photocatalytic activity of H_2_O_2_. Even both Zn^+2^ and ZnO particles have antibacterial activities. The antimicrobial activities of ZnO NPs at nanoscale would yield affordable and safe innovative strategies.^15,49^ Regarding these phenomena and cell responses, the direct interaction between ZnO NPs and cell surfaces can be considered as a reasonable mechanism of ZnO NPs bacterial inactivation.^9,11,35,49^

In comparison with large particles, NPs have more evident antimicrobial activities. The small size (<100 nm) and the high surface-to-volume ratio of NPs facilitate the prerequisite interaction with the microorganisms. As mentioned earlier, antimicrobial activity of NPs such as TiO_2_ and ZnO is usually attributed to its crystal structure, size, shape, and surface area.^9,11,35^ Oxidative stress caused by ROS is particularly the mechanism proposed for NPs. As a result, ROS causes site-specific DNA damage and eventually results in cell death. Although the certain antimicrobial mechanism of NPs has not been well understood, it was revealed that the antimicrobial activity of these NPs has been associated with the following mechanisms: the release of antimicrobial ions, damaging the integrity of bacterial cell in result of interaction of NPs with microorganisms, and the formation of radicals through light irradiation.^9,11,35,48,50^ On the other hand, despite significant potential NPs as antimicrobial compounds, the toxicity of NPs at high concentrations restricts their use in human’s application. Consequently, further studies should be carried out in the future.

## Conclusion

In this study, the kinetic of bacterial death of NPs (TiO_2_ and ZnO) and the susceptibility coefficient were determined and used to evaluate their antimicrobial activity against *L. monocytogenes*, *S. aureus*, *S. enteritidis*, and *E. coli* pathogenic bacteria. Universally, our observations showed that Gram-positive bacterial strains are more sensitive in comparison with gram-negative bacteria against the tested NPs. The effect of NPs suspension on death kinetic of bacteria showed that the survival ratio of bacteria decreased almost linearly with increasing NPs concentration and contact time. The slope (negative) of the survival curve indicates a significant antimicrobial activity of NPs. The sensitivity coefficient and death kinetic can be used to evaluate the antimicrobial effects of various antimicrobial agents. For future studies, the focus should be on the relationship between the antimicrobial activity of these NPs in different sizes with death kinetics and the sensitivity coefficient.

## Ethical Issues

Not applicable.

## Conflict of Interest

The authors declare no conflict of interests.

## Acknowledgments

This study was conducted in Tabriz University of Medical Sciences, Tabriz, Iran.
